# It’s Time for a Change: The Role of Gibberellin in Root Meristem Development

**DOI:** 10.3389/fpls.2022.882517

**Published:** 2022-05-03

**Authors:** Margaryta Shtin, Raffaele Dello Ioio, Marta Del Bianco

**Affiliations:** ^1^Department of Biology and Biotechnology “C. Darwin”, Laboratory of Functional Genomics and Proteomics of Model Systems, University of Rome “Sapienza”, Rome, Italy; ^2^Italian Space Agency, Rome, Italy

**Keywords:** root development, gibberellin, meristem, root meristem, asymmetric division, cell differentiation

## Abstract

One of the most amazing characteristics of plants is their ability to grow and adapt their development to environmental changes. This fascinating feature is possible thanks to the activity of meristems, tissues that contain lasting self-renewal stem cells. Because of its simple and symmetric structure, the root meristem emerged as a potent system to uncover the developmental mechanisms behind the development of the meristems. The root meristem is formed during embryogenesis and sustains root growth for all the plant’s lifetime. In the last decade, gibberellins have emerged as a key regulator for root meristem development. This phytohormone functions as a molecular clock for root development. This mini review discusses the latest advances in understanding the role of gibberellin in root development and highlights the central role of this hormone as developmental timer.

## Introduction

The dynamic development of plants has attracted scientists since Aristotle times (Coren, 2020, Aristotle, Physica). Thanks to the study of meristems, we have now gained a comprehensive knowledge about the basis that governs the time- and space- dependent dynamics of plant development. Among meristems, the root meristem is a great model system because of its simple and symmetric structure, which remains largely invariable for the whole life span of the plant (Di Mambro et al., 2019). In this organ, different developmental stages and cell identities are recognisable by shape and position (Dolan et al., 1993; Scheres et al., 2002). The root meristem can be divided in two distinctive axis: a longitudinal one that spans from the shoot-to-root junction (proximal) to the tip of the root (distal), and a radial one that extends from the centre of the root to the most external tissues (Di Mambro et al., 2019). Longitudinally, at the distal part of the root meristem is the stem cell niche (SCN). The SCN is centred around the organising centre, a small group of slowly dividing cells known as the quiescent centre (QC). Surrounding the QC, and in direct contact with it, are the long-term stem cells (Van den Berg et al., 1997; Di Mambro and Dello Ioio, 2020). These cells generate transit-amplifying (TA) daughter cells, which divide in the division zone (DZ) (Perilli et al., 2012). As plant cells do not migrate due to the presence of a rigid cell wall, proximal dividing TAs push the upper cells toward the adjacent area of the meristem (Dolan et al., 1993; Scheres et al., 1994, 2002). Once the TAs reach the transition zone (TZ), those cells stop dividing and start to elongate exiting from the meristematic zone (Perilli et al., 2012; Di Mambro et al., 2019; Salvi et al., 2020). The number of TAs proliferates in the first phases of development to permit the formation of the meristem to then remain fixed once it reaches the optimal meristem size (Salvi et al., 2020). Radially, the root meristem is formed by concentric rings of tissues where the most internal one is the vasculature, whereas the most external is the root cap (Lee et al., 2013; Di Mambro et al., 2019). Root meristem development is dynamic on both axis (Baum et al., 2002; Scheres et al., 2002; Dello Ioio et al., 2007). The number of tissue layers composing the root meristem increases in time depending also on age-dependent reactive oxygen species accumulation (Cui et al., 2014; Di Ruocco et al., 2018b).

**FIGURE 1:**
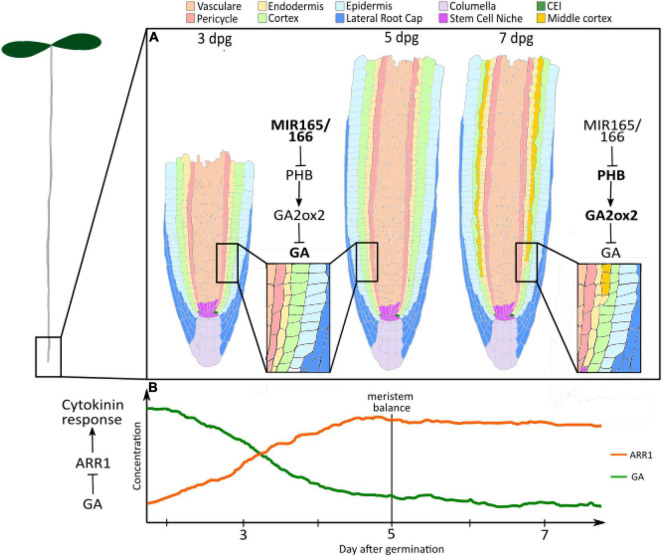
Gibberellin controls longitudinal and radial dynamics. **(A)** GA levels regulate root radial axis patterning: High levels of Gibberellin between 3 and 5 dpg inhibit the formation of the MC. High concentration of miRNA 165 and 166 negatively regulate PHB levels. PHB controls the gibberellin degradation, promoting the expression of the GA2OX2 gibberellin catalytic gene. At 7–8 dpg, the levels of PHB increase as a consequence of miR165 and 166 reduction. PHB reduction results in enhanced GA2OX2 expression, which determines the degradation of GAs and MC formation. Salmon, Vasculature; Pink, Pericycle; Yellow, endodermis; Light green, cortex; cyan, epidermis; blue, lateral root cap; Lilac, columella; Purple, stem cell niche; Green, CEI; Ochre, middle cortex. **(B)** GA levels control root longitudinal axis patterning: Few days after germination, high GA levels activate cell division and repress cytokinin responses through the repression of ARR1 expression. The decline in GA levels from germination to 5 dpg allows for ARR1 expression. This induces an increase in cell differentiation, which balances cell division and sets meristem size. Orange, ARR1; Green, Gibberellin (GA).

In this context, the plant hormone gibberellin has been emerging as a pivotal player in the regulation of root meristem development. With a dynamic but robust biosynthesis cascade and highly integrated signalling pathway, gibberellin seems to have evolved as a molecular switch for temporal variation in growth regimes (Figure 1).

## The Biology of Gibberellin

Gibberellins, or gibberellic acid (GA), are a family of endogenous plant growth regulators (Hedden and Sponsel, 2015). A complex network of biosynthetic and catabolic enzymes regulates the homoeostasis of bioactive gibberellins (GA1, GA3, GA4, and GA7) (Yamaguchi, 2008). In particular, the final limiting steps of gibberellin biosynthesis are catalysed by GA 20-oxidases (GA20OXs) and GA 3-oxidases (GA3OXs) (Chiang et al., 1995; Phillips et al., 1995; Gallego-Giraldo et al., 2008), while GA 2-oxidases (GA2OXs) mediate gibberellin deactivation (Schomburg et al., 2003; Rieu et al., 2008). The genes encoding for the biosynthesis and catabolic enzymes are differentially expressed in specific cells, tissues, and developmental stages, thus mediating the appropriate gibberellin dynamics and distribution (Yamaguchi, 2008). In the root, both *GA3OX* and *GA20OX* genes appear to be expressed in the meristem, in the area closer to the SCN, and in differentiating tissues (Mitchum et al., 2006; Barker et al., 2020). Overall, it would appear that GA biosynthesis takes place in multiple tissues, with the endodermis being a major site of synthesis (Barker et al., 2020). GA catabolism has been shown to take place in the elongation zone (Barker et al., 2020) and in the meristem closer to the SCN (Bertolotti et al., 2021a).

In addition to biosynthesis and catabolism, gibberellin distribution is also affected by long- and short-distance transport (Shani et al., 2013; Regnault et al., 2015; Binenbaum et al., 2018). Like other weakly acidic hormones, GAs are protonated in the apoplast, where the pH is acidic, and therefore able to diffuse through the plasma membrane. Once in the cytoplasm, at a pH∼7.5, GAs become de-protonated and are therefore subjected to an ion-trap mechanism, which limits their ability to diffuse out of cells (Rizza et al., 2021). While no GA efflux carrier has been identified yet, over the past few years, several GA influx transporters have been found (Binenbaum et al., 2018; Rizza and Jones, 2019). Members of the NITRATE TRANSPORTER 1/PEPTIDE TRANSPORTER FAMILY (NPF) proteins have shown to be able to mediate gibberellin uptake by the cell (Chiba et al., 2015; Saito et al., 2015; Kanno et al., 2016). In addition, the members of the SWEET protein family of transporters have been identified as GA importers, both in rice and Arabidopsis (Saito et al., 2015; Kanno et al., 2016; Morii et al., 2020). In the root meristem, the analysis of the distribution of bioactive fluorescent GA forms has revealed that gibberellin transport results in the accumulation of GAs in the endodermis (Shani et al., 2013).

In the cell, GAs are perceived through a simple circuit that, reminiscent of auxin signalling (Del Bianco and Kepinski, 2011) is based on the degradation of a response repressor and subsequent transcriptional regulation of specific genes. GA interaction with its receptor GA-INSENSITIVE DWARF1 (GID1) (Ueguchi-Tanaka et al., 2005) causes a conformational change that allows the GID1-GA complex to bind the members of the DELLA family of GA repressors (Willige et al., 2007). The GID1/GA/DELLA complex is then recognised by the DELLA-specific F-box protein SLEEPY (SLY) that promotes DELLAs ubiquitination and subsequently degradation via 26S proteasome, triggering the GA response (Cheng et al., 2004). The DELLA family is constituted by five members in Arabidopsis: GA-INSENSITIVE (GAI), REPRESSOR OF GA (RGA), RGA-LIKE1 (RGA1), RGL2, and RGL3. Although essential for GA activity and possessing strong transactivation action, DELLA proteins lack a DNA-binding domain. DELLAs act as transcriptional regulators by interacting with other transcription factors that contain DNA-binding domains (Yoshida et al., 2014). DELLAs mediate the crosstalk between GA and light signalling in regulation of cell expansion through the interaction with PHYTOCHROME-INTERACTING FACTORs (PIFs) (Feng et al., 2008; Li et al., 2016). The cooperation with DELLAs negatively influences both PIF activity, by masking their DNA-binding motif, and stability (De Lucas et al., 2008; Feng et al., 2008). In addition, DELLAs positively control the transcription of cytokinin-regulated genes by interacting with type-B ARABIDOPSIS RESPONSE REGULATORs (ARRs) (Moubayidin et al., 2010). This interaction is required for proper root meristem growth and for the onset of skotomorphogenesis (Hauvermale et al., 2012). Moreover, DELLAs have been shown to interact with members of the INDETERMINATE DOMAIN (IDD) subfamily, which in turn belong to the C2H2 type zinc finger superfamily. The DELLA/IDD complex mediates the upregulation of the expression of the GA-positive factor *SCARECROW-LIKE 3* (*SCL3*), which in turn competes with DELLAs for IDD-interaction (Yoshida et al., 2014).

## Gibberellin and the Regulation of the Root Meristem Longitudinal Axis

While an overall positive regulator of meristem size, GAs affect root meristem balance at different levels by regulating different aspects of the cell cycle. As described in the introduction, longitudinally the root meristem is constituted by the SCN at its distal portion, the DZ where TA daughter cells proliferate, and the TZ where TA cells chase to divide and enter the EDZ (Di Mambro et al., 2019). While this overall organisation is established in the embryo, in the few days after germination the root meristem grows in length, defined as the number of cells in the DZ (Dello Ioio et al., 2007; Salvi et al., 2020). This dynamic regulation is due to the opposing effects of the plant hormones auxin, which promotes cell proliferation, and cytokinin, which controls cell differentiation by affecting auxin signalling and distribution (Dello Ioio et al., 2008). In Arabidopsis, the meristematic growth phase chases around 5 days post germination, when an increase in cytokinin signalling balances the auxin input (Dello Ioio et al., 2007; Del Bianco et al., 2013) (Figures 1A,B).

The dynamic regulation of gibberellin is responsible for defining the meristem growth phase. It has been shown that gibberellin levels decline after germination (Moubayidin et al., 2010) (Figure 1B). This correlates with the onset of the expression of the type-B Arabidopsis Response Regulator ARR1, a key regulator of meristem development (Moubayidin et al., 2010; Del Bianco et al., 2013). Indeed, gibberellin application and loss-of-function mutation of *RGA* results in the downregulation of *ARR1* expression, and concomitant lengthening of the meristem (Moubayidin et al., 2010). The decline in gibberellin signalling post germination, therefore, seems to be necessary for allowing *ARR1* expression and meristem balance attainment. Interestingly, the GA-ARR1 module is also targetted by the GRAS-transcription factor SCARECROW (SCR) for the fine regulation of root meristem size. In the endodermis, SCR seems to be able to affect RGA stability by positively regulating the expression of *SNEEZY*, a DELLA-specific F-box (Moubayidin et al., 2016). The molecular mechanisms that determine the decline in GA activity between 3 and 5 dpg are still to be elucidated. Since the decrease in GAs has been inferred from the reduction in the expression of genes involved in GA biosynthesis (Moubayidin et al., 2010), these represent good candidates for the regulation of GA decline. Downstream, the specific transcription factor that interacts with DELLA proteins to regulate the expression of *ARR1* is still to be characterised.

Gibberellic acids have been shown to sustain meristem size by acting as positive regulators of cell division (Ubeda-Tomás et al., 2008, 2009; Achard et al., 2009). Indeed, it has been shown that GA treatment increases cell cycle events in the root meristem, without affecting SCN activity (Ubeda-Tomás et al., 2009). Gibberellins seem to be able to regulate the cell cycle by modulating the expression of the cell cycle inhibitors *KIP-RELATED PROTEIN 2* (*KRP2*) and *SIAMESE* (*SIM*) (Ubeda-Tomás et al., 2009). In this context, it has been suggested that GA/DELLA also regulates proliferation by promoting cell expansion, which is a rate limiting step since cells must double in size before dividing. It has been shown that in the root meristem GA controls cell proliferation specifically from the endodermis (Ubeda-Tomás et al., 2009). Endodermis-targetted expression of a non-degradable mutant version of GAI disrupts root meristem growth and blocks cell proliferation. This is compatible with the observation that GAs are actively transported and accumulated in the endodermis (Shani et al., 2013). Mechanical constraints then transfer this input radially to the other tissues (Ubeda-Tomás et al., 2009). Interestingly, elements of the gibberellin pathway seem to be misregulated in microgravity conditions, which have been shown to enhance cell proliferation but uncoupling it from cell growth (Ubeda-Tomás et al., 2009; Medina and Herranz, 2010). Considering the dual role of gibberellin, it could be argued that gibberellin could play a role in coordinating cell growth and proliferation in normal gravity, a feature that is lost in microgravity.

## Gibberellin Controls the Root Meristem Radial Axis

Root radial axis patterning derives from a coordinated activity of asymmetric cell divisions occurring in both the stem cell daughters and TA cells (Scheres et al., 1994, 2002; Di Ruocco et al., 2018a). For example the ground tissue, a tissue composed by endodermal and cortical layers, derives from asymmetric cell divisions occurring in an initial daughter (Scheres et al., 1994; Di Ruocco et al., 2018a). The ground tissue patterning is an optimal model system to understand the mechanisms coordinating patterning in time and space. Radially, the ground tissue is formed by cortical layer(s) and endodermis (Di Ruocco et al., 2018a). Differently from most of the plant species, in Arabidopsis only one cortical layer is formed during embryogenesis (Scheres et al., 2002; Di Ruocco et al., 2018a). However, in about 80% of Arabidopsis plants a second cortical layer, called Middle Cortex (MC), enriches the root meristem patterning between 7 and 8 days post germination (Baum et al., 2002; Cui and Benfey, 2009; Choi and Lim, 2016; Figure 1A). The origin of the MC differs from the cortical layer one. Whereas the cortical layer is generated by the periclinal asymmetric division of the Cortex and Endodermis Initial Daughter (CEID), the MC derives from asymmetric divisions of the endodermis (Scheres et al., 1994, 2002; Di Ruocco et al., 2018b; Figure 1A). Gibberellin has been shown to play a central role in the timing’s control of MC formation (Paquette and Benfey, 2005; Moubayidin et al., 2010).

High levels of gibberellin during early root development inhibit the formation of the MC, whereas a later reduction of gibberellin quantity promotes it (Paquette and Benfey, 2005; Cui and Benfey, 2009; Gong et al., 2016; Lee et al., 2016; Di Ruocco et al., 2018a). DELLA proteins RGA and GAI mediates GA activity in MC formation (Paquette and Benfey, 2005; Bertolotti et al., 2021a; Figure 1A). Indeed, loss of function mutants of *RGA* and *GAI* show a lower amount of plants producing the MC, since GA insensitive versions of these show premature MC formation (Paquette and Benfey, 2005; Bertolotti et al., 2021a). GAI activates the expression in the endodermis of the cell cycle regulator *CYCLIN D6;1* (*CYCD6;1*), promoting the asymmetric cell division in those cells. Nonetheless, the molecular mechanisms allowing GAI to promote this cell cycle regulator are still unknown. It has been recently shown that variation in gibberellin levels depends on the activity of the HOMEODOMAIN-LEUCINE ZIPPER III (HD-ZIPIII) transcription factor PHABULOSA (PHB) (Bertolotti et al., 2021a; Figure 1A). Indeed, PHB controls the gibberellin degradation promoting the expression of the *GA2OX2* gibberellin catalytic gene (Bertolotti et al., 2021a). *PHB* expression increases during root maturation, incrementing the gibberellin degradation and, thus, promoting GAI stability (Bertolotti et al., 2021a,b). The dynamic expression pattern of *PHB* depends on the reduction of the expression of the HD-ZIPIII repressors *microRNA 165* and *166*, whose quantity decreases at later stages of root development (Bertolotti et al., 2021b; Figure 1A). The molecular mechanisms controlling the time dynamics of miR165 and 166 are still vague. Since the GRAS transcription factors SHORTROOT (SHR) and SCARECROW (SCR) activate transcription of these miRNAs, it might be the case that those transcription factors show a temporal dynamic expression pattern (Carlsbecker et al., 2010; Miyashima et al., 2011; Bertolotti et al., 2021b). Other than activating *miR165* and *166* expressions, SHR and SCR together with SCL3 play a fundamental role in regulating the formation of the MC. SCR and SCL3 together with high levels of SHR protein inhibit MC formation, whereas low levels of SHR in the endodermis are required for activating *CYCD6;1* expression in the endodermis. GAs antagonise SHR, SCR, and SCL3 activity regulating SHR abundance negatively acting on the SEUSS protein, an elicitor of SHR, SCR, and SCL3 transcription and promoting DELLA degradation, whose activity is required for *SCL3* expression (Paquette and Benfey, 2005; Heo et al., 2011; Bertolotti et al., 2021a; Zluhan-Martínez et al., 2021).

Genetic experiments suggest that SHR/SCR/SCL3 and PHB promote the MC formation independently (Di Ruocco et al., 2018a). However, those two pathways might crosstalk on GA regulation. Future research will clarify this point.

## Conclusions

Gibberellic acids control different developmental processes: from seed germination to organ elongation, from flowering to fruit development (Pacifici et al., 2015). In this review, we have shown how, in the root, GA signalling integrates with that of many other pathways to dynamically regulate radial and longitudinal patterns. From an evolutionary point of view, the initial gibberellin regime that allows for the longitudinal expansion of the root meristem could allow for the plant to increase root growth after germination to rapidly anchor the plant to the soil and secure vital resources such as water and nutrients. Recent interesting evidence demonstrates that GAs are involved in the cortex proliferation of the leguminose *Medicago truncatula* (Fonouni-Farde et al., 2019), supporting the idea for a role of this hormone in the promotion of the diversity in root patterning among plants. PHB, SHR, and SCR have been shown to be fundamental players for interspecific variability in cortical layer variability (Cui et al., 2007; Wu et al., 2014; Henry et al., 2017; Di Ruocco et al., 2018a; Ortiz-Ramírez et al., 2021). Considering their described role in the control of GA homoeostasis, it might be the case for a crosstalk between those elements and GA in controlling the variability of root morphologies.

In this review, we focussed on the root meristem primary axis, but GAs have been shown to play a role in other dynamic growth responses like gravitropism, i.e., the ability of a plant to adapt their post-embryonic development according to their position in the gravity vector. A widely conserved trait, this response is a key component of regulating plant architecture. Root graviresponse starts in the columella cells, where the sedimentation of starch-filled plastids (amyloplasts), according to the gravity vector, triggers the repolarisation of the auxin transporters PIN-FORMEDs to the lower side of the cell (Su et al., 2017). Consequently, auxin accumulates in the lower half of the root, where it inhibits cell elongation, thus triggering bending of the root tip. The involvement of GAs in the gravitropic response appear to be conserved in the entire plant kingdom, from mosses (Vandenbussche et al., 2007) to higher plants (Löfke et al., 2013), from grasses (Wolbang et al., 2007) to trees (Lopez et al., 2021). In Arabidopsis, it has been shown that GA distribution and response are asymmetric in the graviresponding root, with the maximum at the lower side of gravistimulated roots. This seems to correlate with the increase in membrane-localised auxin transporter PIN-FORMED 2 (PIN2) (Löfke et al., 2013) in the lower half of the root, which has been suggested to be important to increase the robustness of the gravitropic response (Paciorek et al., 2005; Abas et al., 2006). Conversely, in the shoot, it has been suggested that the interaction between auxin and GAs in hypocotyl gravitropism maintains a high degree of flexibility in tropic responses (Gallego-Bartolomé et al., 2011).

Gibberellins seem to have evolved as fine regulators of the dynamic development of plants. It could be argued that this role could have its roots in the peculiar characteristics of gibberellins: from their tightly feedbacked biosynthesis pathway (Ross, 1994), to the possible non-specific, albeit not fully characterised, transporting system (Rizza and Jones, 2019), and a transcriptional response that does not rely on GA-specific transcription factors. These features make gibberellin a good candidate as an interaction hub for different signalling pathways for a robust integration of complex developmental responses. Future studies to decipher the molecular details of gibberellin signalling and transport will clarify this possibility.

## Author Contributions

MS, RD, and MD conceptualised and wrote the manuscript, and prepared the figures. All authors contributed to the article and approved the submitted version.

## Conflict of Interest

The authors declare that the research was conducted in the absence of any commercial or financial relationships that could be construed as a potential conflict of interest.

## Publisher’s Note

All claims expressed in this article are solely those of the authors and do not necessarily represent those of their affiliated organizations, or those of the publisher, the editors and the reviewers. Any product that may be evaluated in this article, or claim that may be made by its manufacturer, is not guaranteed or endorsed by the publisher.
